# A Structural Basis for Cellular Uptake of GST-Fold Proteins

**DOI:** 10.1371/journal.pone.0017864

**Published:** 2011-03-24

**Authors:** Melanie J. Morris, Dan Liu, Llara M. Weaver, Philip G. Board, Marco G. Casarotto

**Affiliations:** The John Curtin School of Medical Research, Australian National University, Canberra, Australian Capital Territory, Australia; University of South Florida College of Medicine, United States of America

## Abstract

It has recently emerged that glutathione transferase enzymes (GSTs) and other structurally related molecules can be translocated from the external medium into many different cell types. In this study we aim to explore in detail, the structural features that govern cell translocation and by dissecting the human GST enzyme GSTM2-2 we quantatively demonstrate that the α-helical C-terminal domain (GST-C) is responsible for this property. Attempts to further examine the constituent helices within GST-C resulted in a reduction in cell translocation efficiency, indicating that the intrinsic GST-C domain structure is necessary for maximal cell translocation capacity. In particular, it was noted that the α-6 helix of GST-C plays a stabilising role in the fold of this domain. By destabilising the conformation of GST-C, an increase in cell translocation efficiency of up to ∼2-fold was observed. The structural stability profiles of these protein constructs have been investigated by circular dichroism and differential scanning fluorimetry measurements and found to impact upon their cell translocation efficiency. These experiments suggest that the globular, helical domain in the ‘GST-fold’ structural motif plays a role in influencing cellular uptake, and that changes that affect the conformational stability of GST-C can significantly influence cell translocation efficiency.

## Introduction

**Figure 1:**
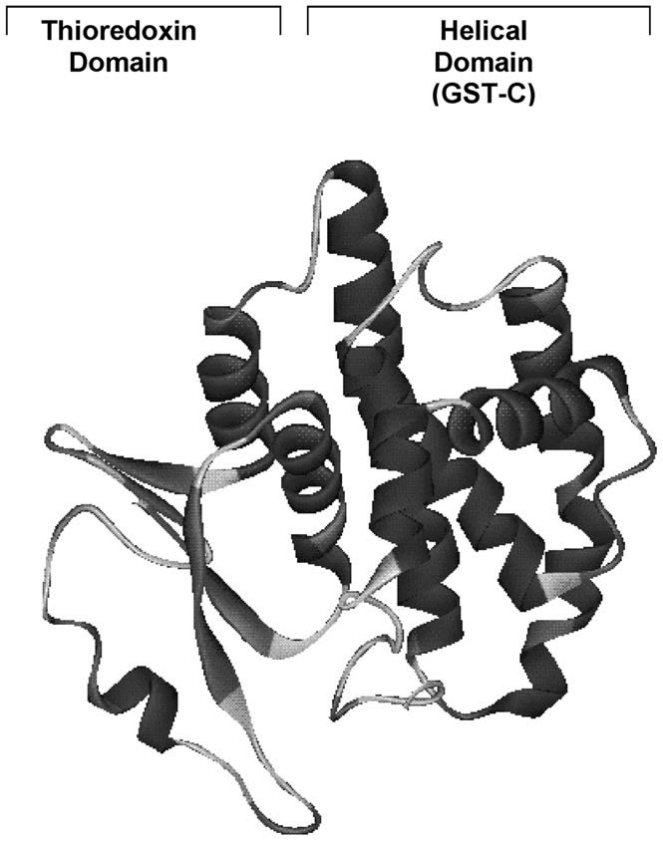
Structural GST-fold. Ribbon diagram demonstrating the GST-fold structure of GSTM2 complexed to 2,4-dinitrophenyl glutathione (not shown in figure). Note the N-terminal thioredoxin fold and C-terminal α-helices. The figure was generated by use of the DS modelling 1.1 program (Accelrys) from the accession bank file RCSB-1XW5. The arrow denotes the cleavage site (residue 88) that separates the two domains.

Glutathione transferases (GSTs) are an important family of enzymes that participate in detoxification reactions by conjugating the tripeptide glutathione (GSH) to a wide range of electrophilic and generally hydrophobic compounds. By doing so, toxic, non-polar molecules are rendered more water soluble and are ultimately exported from the cell through ATP-dependent Phase III transporters such as the multidrug resistance associated proteins [1]. GSTs can be broadly divided into at least three categories that include the soluble cytoplasmic GSTs, the microsomal bound GSTs and a mitochondrial GST. The soluble cytoplasmic GST family is widespread across all organisms and consists of a large number of enzymes that can be further characterised into classes. Despite the relatively low sequence homology between some GST classes, all cytosolic GSTs share the same general structure – an N-terminal thioredoxin fold motif and a strongly helical C-terminal domain as shown in Figure 1. It must be noted that in addition to the GST family of enzymes, several other proteins which do not possess GST enzymic activity, are known to display the same structural fold (GST-fold) [2], [3].

In an unexpected finding, it has been reported in independent studies that the *Schistosoma japonicum* glutathione transferase (Sj.GST26) effectively enters cells through an energy-dependent process involving endocytosis [4], [5]. Furthermore, it was found that this phenomenon extended beyond Sj.GST26 to other classes of GST proteins as well as to proteins lacking GST enzyme activity but possessing a GST-fold. It was therefore proposed that the GST structural fold played some undefined role in cell entry [4]. The overall aim in the current study is to strategically dissect a typical human GST enzyme (GSTM2-2) and investigate which structural elements are responsible for its cell translocation properties. By targeting specific amino acid residues we aim to test the hypothesis that the GST-fold is responsible for cell translocation of these molecules. Our investigation revealed that entry of the C-terminal domain of GSTM2 (GST-C) into cells is responsible for cell translocation. Furthermore, by targeting the α6 helix of GST-C by site-directed mutagenesis we showed that significant gains in cell entry were obtained by de-stabilising the protein structure.

## Results

Proteins possessing a GST-fold structure have previously been shown to be capable of efficiently transfecting various cell lines and tissue types [4]. Within this structural protein family the CLIC2 protein was shown to possess the greatest translocation efficiency, while the GSTM2 protein along with several other GST family members displayed marginally less but distinct cell translocation properties. The GSTM2 protein was chosen as a representative GST due to its stability, ease of expression/purification and for its ability to accommodate amino acid modifications [6], [7]. In this study we have investigated the structural characteristics of GSTM2 that govern the efficiency of its translocation into L929 cells.

### The C-terminal domain of GSTM2 is responsible for cell translocation

**Figure 2:**
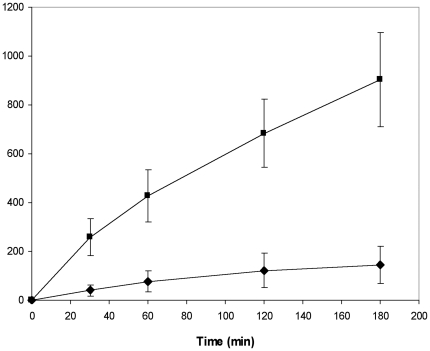
Cellular uptake of GSTM2-2 full length protein compared with its C-terminal domain (GST-C). L-929 cells were incubated with 200 nM GSTM2-OG (◆) or 200 nM GST-C-OG (■) for the indicated time periods and intracellular fluorescence measured by flow cytometry. The mean cell fluorescence of each sample was normalised for the degree of fluorescence labeling of that protein. The data represent the mean ± SD of three independent experiments.

As seen in Figure 1, GST-fold proteins are comprised of an N-terminal thioredoxin fold motif and a strongly helical C-terminal domain. It was of initial interest to determine whether the cell translocation properties of GSTM2 could be attributed to one of these domains. In order to accurately assess the uptake of the individual N and C-terminal domains of GSTM2, these fragments were recloned into the pHUE vector [8], expressed and purified. Unfortunately, the N-terminal thioredoxin fold proved to be insoluble during expression, however the C-terminal α-helical domain (GST-C) was efficiently expressed and purified. The efficiency of cell entry of the Oregon Green labeled GSTM2 protein (GSTM2-OG) and its labeled C-terminal fragment (GST-C-OG) are compared in Figure 2. Surprisingly, the level of internalisation of GST-C exceeded that of the full length protein by a factor of at least four over a three hour period.

**Figure 3:**
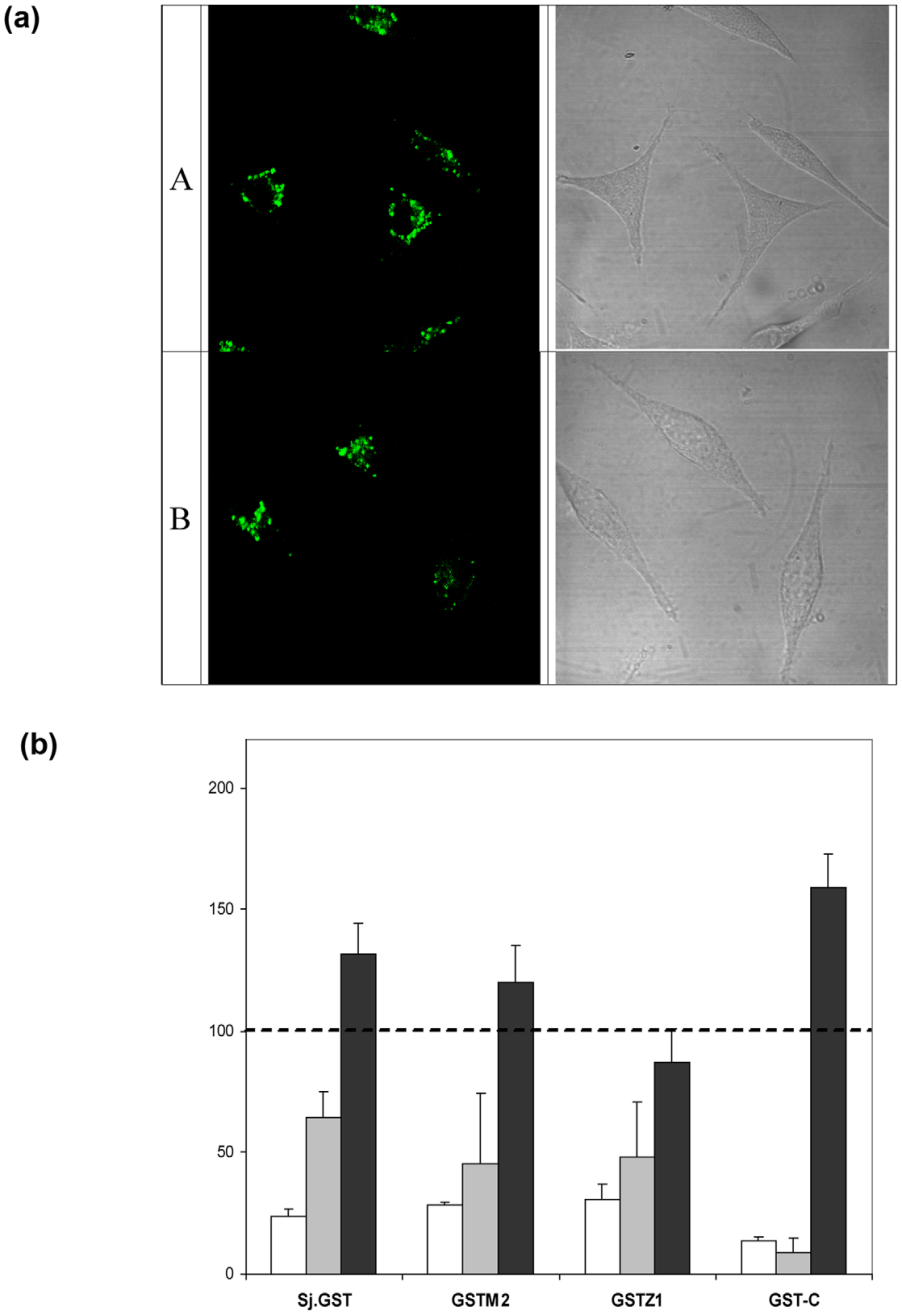
GST-C and GSTM2 proteins show comparable localisation and endocytosis inhibition profiles in L-929 cells. (a) L-929 cells were incubated for 1 hr with 200 nM GSTM2-OG (A) and 200 nM GST-C-OG (B), washed and then observed by confocal microscopy. The fluorescence from both GSTM2 and GST-C is in a punctate pattern in the cytoplasm, whilst minimal cell fluorescence is observed from BSA. (b) Impact of pathway-specific endocytosis inhibitors on GST-C uptake. L-929 cells were incubated for 2 hrs with 200 nM GST-C alongside GSTM2-OG, GSTZ1-OG and Sj.GST-OG after inhibition of endocytosis pathways by 8 µg/mL chlorpromazine (white), 5 mM amiloride (light grey), or 10 µg/mL filipin (dark grey). The impact of inhibitors on GST internalisation was measured quantitatively by flow cytometry. Data is presented as the percentage intracellular fluorescence in treated cells compared to intracellular fluorescence in the absence of inhibitors (dashed line represents uninhibited control). Error bars represent the SD of three independent experiments.

To investigate whether the translocation of GST-C utilises the same mechanism of cell entry as its parent molecule, the uptake of GST-C-OG was initially compared by confocal laser scanning microscopy. After a one hour incubation period, images of the Oregon Green labeled GSTM2 (Panel A) and GST-C (Panel B) revealed a similar punctate pattern throughout the cytoplasm of cells (Figure 3a). Control experiments using Oregon Green labelled BSA revealed no cell uptake (data not shown). Further investigation of the mechanism of GST-C translocation was performed by measuring cell uptake after treatment of cells with the known endocytotic inhibitors chlorpromazine, amiloride and filipin. Chlorpromazine dissociates the clathrin lattice from coated pits, amiloride prevents macropinosome membrane ruffling and filipin restricts lipid raft and caveolae endocytosis [9], [10], [11]. Figure 3b displays the results of endocytic inhibition of GST-C-OG uptake alongside that of GSTM2-OG as well as other representative GST enzymes Sj.GST-OG and GSTZ1-OG. GST-C translocation clearly follows the same trends as that of the full-length GSTs, with chlorpromazine and amiloride having a negative effect on the level of uptake, whilst filipin had a positive effect. However, the degree of inhibition to GST-C translocation caused by amiloride was considerably greater than that imposed on the full-length GSTs suggesting that macropinocytosis may have a more pronounced role in the translocation of the C-terminus.

**Figure 4:**
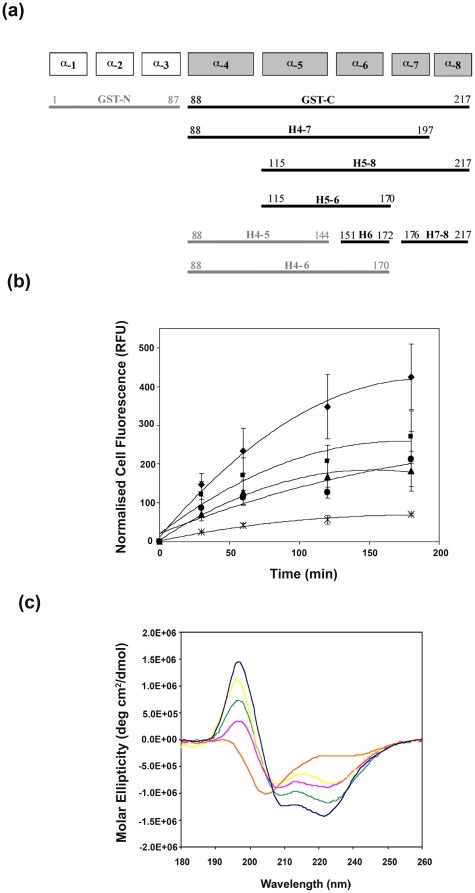
Structural and cell translocation analysis for α-helical fragments derived from GST-C. (a) Schematic diagram outlining α-helical fragment constructs of GSTM2. Peptides H6 and H7–8 were synthesized. The peptides in bold denote fragments that have been tested. (b) Comparative cellular uptake of GSTM2 C-terminal peptides. L-929 cells were incubated with 200 nM OG-labelled peptides, GSTM2 protein (X), GST-C (◆) and H4–7 (■), H7–8 (●), H5–8 (▲) fragments of GST-C for the indicated time periods and intracellular fluorescence measured by flow cytometry. The mean cell fluorescence of each sample was normalised for the degree of fluorescent labeling of that protein. Data represents the average of triplicate measurements from three independent experiments ± SEM. (c) Circular dichroism spectra for the full-length GSTM2 protein (dark blue) versus GST-C (green) and H4–7 (cyan), H7–8 (orange), H5–8 (pink) fragments of GST-C. All proteins/peptides were measured at 4–5 µM concentration in 10 mM sodium phosphate buffer pH 7.2.

Given the accumulating evidence that GST-C is the domain responsible for cell translocation, the structure of the GSTM2 C-terminus was further shortened and these constructs examined for their cell translocation efficiency. A series of constructs were designed to include different helical segments present in the full-length GSTM2 crystal structure (PDB-1XW5). Figure 4a shows a summary of the helical fragments which retained solubility after recombinant expression and purification, in addition to two shorter synthesized peptides. Because of the smaller size and fewer available lysine and arginine residues, the shorter C-terminal peptides were not amenable to amine-labeling with Oregon Green, and only the larger, multi-helical peptides, H4–7, H5–8 and H7–8 achieved a satisfactory dye-to-protein ratio. To verify the translocation of these fragments, flow cytometry was performed on the fluorescently-labeled forms. The two largest C-terminal fragments (H4–7 and H5-8) had a lower translocation rate than GST-C over 3 hrs (Figure 4b) and the translocation capacity of the H7–8 peptide was diminished still further. The viability of all fragment-treated cells was not compromised at these concentrations, as judged by membrane permeability to 7-AAD.

Circular dichroism was employed to ascertain the overall secondary structure (particularly the helical content) of the C-terminal domain and its peptide fragments. Figure 4c confirms that the strong helical component present in the GSTM2 full-length protein is retained by the GST-C as well as peptides H4–7 and H5–8 – note the varying spectra amplitudes reflect differences in peptide sizes and not structural content. In contrast, the H7–8 peptide has a very different structure to the other fragments and appears to be disordered in nature. The CD spectra of synthesized peptide fragments corresponding to the helix 4 and helix 6 sequences were also found to be unstructured (data not shown) indicating that the individual helical fragments are not sufficient to adopt α-helical structures.

### Structural stability of GSTM2-2 and GST-C fragments

**Figure 5:**
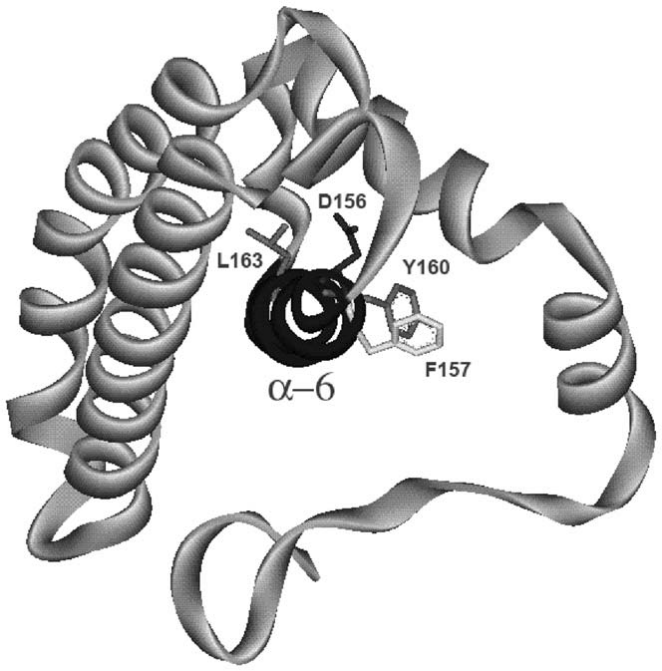
Ribbon structure model of the C-terminal domain of GSTM2, taken from the crystal structure of the full-length protein – PDB file 1XW5. The α-6 helix is highlighted in bold and is surrounded by other α-helical elements of GST-C. Residues mutated to probe the role of the α-6 helix in cell translocation are displayed and labeled.

The data so far suggests that the overall structure of the GST C-terminus may be important for optimal cell uptake. Visualisation of a ribbon structure model of GST-C (based on the x-ray crystallography structure of the whole protein) reveals that the α-6 helix forms the hydrophobic core of this domain and is surrounded by helices 4, 5, 7 and 8, and connected through a series of ionic and hydrophobic interactions (Figure 5). This overall helical configuration is also strikingly evident in structural models of the other GST-fold superfamily proteins, including CLIC2 which has been noted to display comparable structural features to α pore-forming toxin proteins [12]. This configuration seems to suggest that H6 could play a central role in the translocation of this class of molecule.

**Figure 6:**
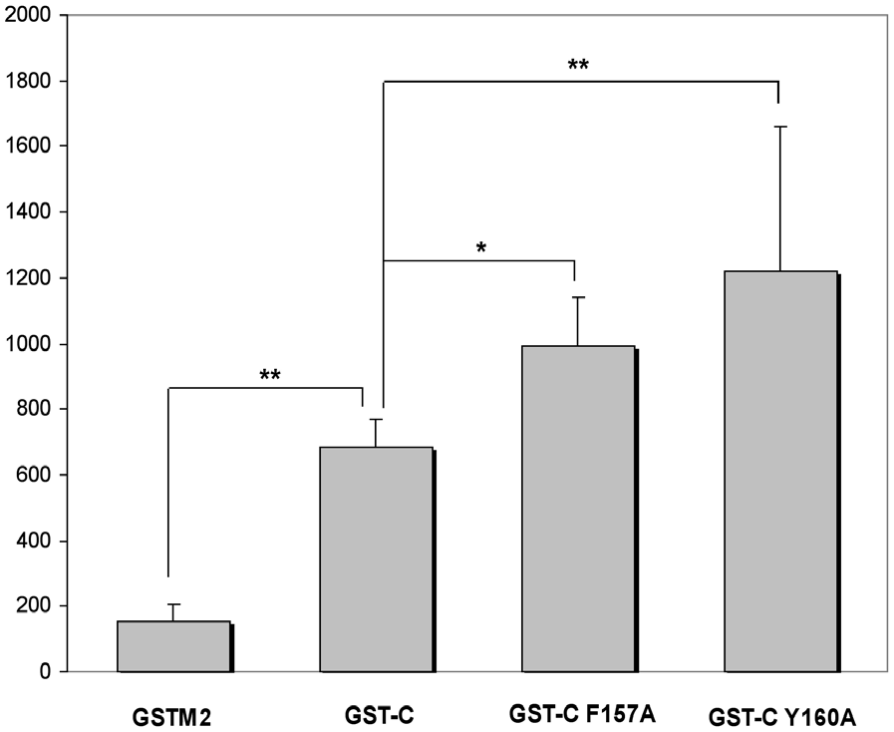
Effect of substitution mutations within the GSTM2 α-6 helix upon cellular translocation of the C-terminal domain (GST-C). L-929 cells were treated for 2 hours with 200 nM Oregon Green-labelled GST-C variants and GST-C wildtype. The mean cellular fluorescence of each sample was normalised for the degree of fluorescent labeling of that protein. Data represents the average of four to six independent experiments ± SEM. Significantly changed translocation efficiency (as determined by student's paired t-Test; one-tailed) is indicated by an asterisk (* = P<0.05; ** = P<0.01).

To test this possibility, we aimed to disrupt key contacts between the α-6 helix and surrounding helices, and monitor changes in cell uptake efficiency. By analysing the X-ray crystallography structure of the entire GSTM2 protein (RCSB-1XW5), four residues within the α-6 helix were initially identified as being in close proximity (<5 Å) to partner amino acid residues located in helices 5, 7 and 8, thereby potentially participating in hydrogen-bond, electrostatic or hydrophobic interactions. Four modified GST-C proteins were produced by mutating each of the selected residues (Y160; F157; L163; D156) to an alanine residue with the specific aim of disrupting these contacts. Figure 5 displays the ribbon structure of the C-terminal domain with the position of all mutated residues highlighted. The D156A mutant could not be recombinantly expressed, and the L163A mutant expressed only in low quantities and was highly unstable in solution, suggesting that the conformation of the α-6 helix is a key element in the folding and hence the stability of the C-terminal domain. The contact residues for the D156 side chain are F147 and Thr153 (amide backbone) while for L163 hydrophobic contacts are made with the side chains of F103 and F183. However, the Y160A and F157A were successfully purified to levels that enabled fluorescence labeling. To monitor the effect of these structural mutations on the translocation of the GSTM2 C-terminus, Oregon Green labeled variants and wildtype GST-C were incubated with L-929 cells, and the amount of protein internalised after two hours measured by flow cytometry. The translocation efficiency of each variant relative to that of the wildtype GST-C is shown in Figure 6. The two destabilising mutations, F157A and Y160A, resulted in greater uptake of the C-terminus by approximately 45 and 85%, respectively, indicating that these structural contacts to the α-6 helix are significant for GST translocation. To explore whether the mutations made to the C-terminus protein sequence produced a change in secondary structure, circular dichroism was performed on all C-terminal variants (data not shown). At room temperature no discernable change in secondary structure was observed between the F157A, Y160A variants and wildtype GST-C.

**Figure 7:**
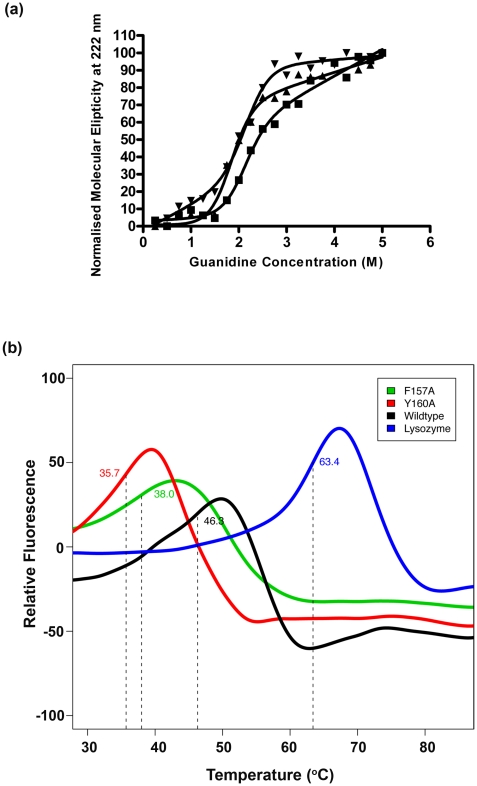
Stability studies of GSTM2-2 and GST-C variants measured by circular dichroism (CD) and differential scanning fluorimetry. (a) Normalised molecular ellipticity at 222 nm as a function of guanidine HCl concentration (0–5 M) as measured by CD experiments, GST-C (■), Y160A (▼) and F157A (▲). (b) Differential scanning fluorimetry profiles of GST-C wildtype (black), GST-C/Y160A (red), GST-C/F157A (green) and hen egg-white lysozyme control (blue). Melting temperatures (T_m_) are denoted by vertical broken lines.

Even though there were no substantial secondary structure differences observed between GSTM2, the GST-C or the mutated GST-C molecules, this does not necessarily signify that the mutations/truncations made to GSTM2 are structurally inert. Given that the mutants were engineered to abolish key protein intra-molecular interaction, it is highly probable that the modifications could lead to changes in the structural stability of these GST-C variants. To test this possibility we performed denaturing experiments using circular dichroism (CD) and differential scanning fluorimetry (DSF) techniques. For CD experiments, the secondary structure profiles of GST-C and its mutants were monitored in the presence of varying concentrations (0–5 M) of the denaturant guanidine HCl. In this case, the molar elipticity at 222 nm (an indicator of α-helical structure) was measured as a function of guanidine concentration and presented in Figure 7a. It has been previously noted that for some dimeric proteins (including GSTs) that an intermediate unfolding state is detectable when a three-state unfolding model is applied [13], [14], [15]. It is not clear whether this is the case for GST-C or its mutants since fitting of the data using a three-state unfolding model or a two-state model were of similar quality. Using the two state-model eqn 1 [16] (experimental) the transition state parameters of the three GST-C variants were obtained (Table 1). The free energy of unfolding was greatest for the GST-C wild-type construct followed F157A and Y160A.

**Table 1:** Thermodynamic parameters characterising the guanidine-induced unfolding transition of GST-C and the mutants F157A and Y160A.

GSTM2-2 Variant	ΔG(kcal/M)	m(kcal/M)	[X]_half_(M)
GST-C	5.20±0.88	2.50±0.39	2.08±0.04
Y160A	4.87±0.72	2.68±0.35	1.82±0.03
F157A	4.95±0.59	2.31±0.24	2.15±0.03

Measurements were performed by monitoring the molar ellipticity at 222 nm (CD) using guanidine HCl (0–5 M) as a denaturant.

The stability of these proteins was also assessed by DSF by comparing the melting temperatures of the GST-C and the Y60A, F157A mutants. These mutants have a negative effect upon stability with the Y160A mutant showing the lowest meting temperature at 35.7°C followed by the F157A mutant at 38.0°C. The GST-C protein shows a melting temperature of 46.3°C while the control protein, hen egg white lysozyme (which contains four intramolecular disulphide bridges) has the highest T_m_ at 63.4°C. Combined with the CD denaturation data we can conclude that F157 and Y160 participate in stabilising the C-terminal domain of GSTM2. Notably, the degree of protein stability is inversely proportional to the capacity of the GST-C protein to enter cells.

## Discussion

### The C-terminus of GSTM2-2 drives cell translocation

It is apparent from these studies that the driving force for GST cell translocation resides with the α-helical C-terminal domain rather than the entire GST-fold structure itself. This conclusion is borne out of the domain studies where the C-terminal domain of GSTM2 (GST-C) was found to be approximately four fold more efficiently translocated in L929 cells compared to the full protein. Although we were unable to express and test the thioredoxin domain separately, an investigation by Namiki et al [5] found that *E.coli* thioredoxin was not translocated into cells, suggesting that the thioredoxin domain of Sj.GST26 and by inference the thioredoxin domain of GST-fold proteins in general may not be responsible for cell translocation [5]. Unlike protein transduction domains whose core translocating properties can often be ascribed to short ‘cell-penetrating’ peptides [17], [18], [19], the most efficient translocation module of GST proteins appears to be the C-terminal domain in its entirety. Attempts to minimize the size of the GST-C to smaller fragments that are still capable of equivalent rates of cell uptake revealed a reduced capacity for cell translocation. Therefore in the case of GST-C, it seems that all the helices of the C-terminus act in a concerted manner to promote cell translocation. The mechanism of cell translocation remains unclear but does involve endocytosis [4] and is likely to take place through an interaction with a cell-surface receptor and/or by insertion into the cell lipid bilayer. By removal of the thioredoxin domain from GSTM2, it is possible that recognition of a cell surface receptor is enhanced, or equally, the structural changes within the C-terminal helices may facilitate more effective membrane insertion.

An important finding arising from this study is that the core structure of the GST C-terminal domain is essential for cell translocation, with the hydrophobic helix α-6 playing a structurally central role. The topology of the GST-C domain belongs to the well-known globin family and the specific orientation of the α-helices forms a particular subset within this family where the two layers of helices sit almost orthogonal to each other [20] (see Figure 5). Alpha pore-forming toxins such as endotoxins, colicins, and diphtheria toxin [21], [22], [23] are members of this family and it has been proposed that the membrane-penetrating domains of these proteins contains a buried, hydrophobic helical hairpin structure which inserts into the lipid bilayer of cell membranes (or endosomal membrane in the case of diphtheria toxin) and facilitates pore formation [24], [25]. Such a structure is also present within some members of the Bcl family of apoptosis-regulating proteins, although whether membrane insertion correlates with their pro-apoptopic function is not currently clear [26], [27], [28]. It has been noted that the low pH environment associated with the membrane surface may play a role in promoting cell insertion of the colicins, diptheria toxin and Bcl-X_L_ [26].

It has been previously suggested by Cromer et al [12] that one potential mechanism of cell association by CLIC proteins - proteins that possess a GST-fold - involves the insertion of hydrophobic helix-6 into the cell membrane as part of a pore-forming process. All GST-fold proteins contain at least one hydrophobic helix surrounded by a bundle of other helices in their C-terminal domain. This single helix may be sufficient to interact with cell membranes and thereby directly enhance the rate of endocytosis of these proteins, or alternatively enable greater access to a membrane receptor capable of promoting endocytosis. It is also possible that this hydrophobic helix forms a hairpin structure in conjunction with a neighbouring helix (α-5 or α-7 in the case of GST-C), creating a structural feature common to the aforementioned toxins.

### Decreased stability within GST-C enhances cell translocation

By modifying the structural elements within GSTM2 we have demonstrated a capacity to affect the level of cell translocation. This is evident on a number of fronts. It was previously noted that substitution of key catalytic amino acids in GSTM2 (Y7F), GSTA1(Y9F) and GSTO1 (C32A) resulted in significant increases (up to four fold) in cell translocation efficiency [4]. These mutations, which are located in the thioredoxin domain of the enzymes, not only served to inactivate these enzymes but we suggest also initiate structural and dynamic changes that are transmitted across to the enzyme C-terminal domain. This idea is supported by crystallographic evidence where apo and holo (GSH) GSTA1-1 structures show large structural and dynamic differences in the α-helical C-terminal domain, despite the fact that GSH binds primarily through the thioredoxin domain [29]. We propose that the removal of the thioredoxin domain of GSTM2 also has structural and dynamic effects upon the C-terminal domain of GSTM2 which is reflected in a ∼four fold increase in cell translocation efficiency.

A set of experiments designed to test the idea that structural instability affects the translocation efficiency of GST-C was performed by mutating two residues in helix α-6 in GST-C (F157A and Y160A). By altering these key, targeted residues within GST-C we were able to demonstrate a considerable increase in cell translocation efficiency. Moreover, for this set of three proteins, we were able to correlate the efficiency of cell translocation with protein stability as judged by CD denaturing and differential scanning fluorimetry experiments.

In conclusion we have demonstrated that the structural element responsible for GSTM2 cell translocation is the C-terminal globin-like domain and that gains in cell translocation efficiency may be achieved by altering the conformational stability of this domain. We have shown that the conserved globular fold of this domain which is found in all GST-fold proteins displays a remarkable structural similarity to the α pore-forming toxin domains. What remains to be resolved is whether the mechanism of cell entry of these two functionally unrelated protein classes are alike, an issue that will be pursued in future studies.

## Methods

### Materials

All cellular inhibitors were purchased from Sigma. Oregon Green 488 carboxylic acid succinimidyl ester ‘5-isomer’ was from Molecular Probes and 7-amino-actinomycin D (7-AAD) was from BD PharMingen.

### Expression and Labeling of Recombinant Proteins

GSTM2-2 was expressed in *E. coli* and purified by GSH affinity chromatography as previously described [6]. The cDNA encoding the fragments of GSTM2-2 were amplified by PCR and cloned in-frame downstream of a poly-histidine-tagged ubiquitin sequence in the plasmid pHUE. Protein was purified and the ubiquitin tag cleaved as previously described [8]. In the accepted nomenclature [30] GSTM2-2 refers to the dimer of this protein. However in the interest of simplicity, the enzyme will be referred to as GSTM2 hereafter.

Purified proteins were dialysed into PBS prior to fluorescent labeling of primary amines with Oregon Green succinimidyl ester according to manufacturer's instruction. Labeled proteins were passed through size-exclusion sephadex columns then dialysed for 48 hours against PBS at 4°C to ensure efficient removal of free dye. Protein concentration and dye to protein ratios were calculated from protein absorbance at 280 nm and 496 nm according to the manufacturer's labeling protocol. All Oregon Green-labeled proteins were aliquoted and stored at −20°C. Synthesized peptides were resuspended in PBS and labeled following the same procedure. To distinguish labeled proteins the suffix –OG has been added to the nomenclature i.e. GSTM2-OG.

### Cell Culture

L-929 mouse fibroblast cell line was obtained from ATCC and routinely maintained in RPMI 1640 medium, supplemented with 10% fetal bovine serum (FBS) (v/v), 2 mM glutamine and 2 g/L NaHCO_3_ at 5% CO_2_ and 37°C. Cultures were passaged using PBS containing 0.05% trypsin and 0.02% EDTA (v/v). All cell culture reagents were purchased from Gibco.

### Flow Cytometry

To quantitatively investigate the cellular uptake of GSTM2 and its fragments, as well as the effect of endocytosis inhibitors upon cell uptake, L-929 cells were seeded at a density of 8×10^4^ per well in 12-well plates (Nunclon) in 10% FBS/RPMI 1640 medium. After 24 hrs the cells were rinsed with serum-free RPMI 1640 medium before addition of inhibitors or fluorescently-labelled proteins in serum-free medium. Following protein incubation, cells were washed several times with PBS and detached by trypsinization for 10 minutes at 37°C. Cells were centrifuged at 4°C, washed in cold PBS containing 2% FBS (v/v) then resuspended in 2% FBS/PBS containing 0.5 ng/µL 7-amino-actinomycin D (7-AAD) in order to label nuclei of membrane-damaged cells. Cells were incubated at room temperature in the dark for 10 minutes prior to fluorescence-activated cell sorting of 10^4^ counts on a FACScan flow cytometer (Becton Dickinson). Cells with 7-AAD fluorescence were considered nonviable and excluded from histogram acquisition, and the geometric mean fluorescence of the viable population was used for standardization. To compare cellular uptake of different proteins, geometric mean fluorescence values were divided by the dye to protein ratio to give a normalised value independent of the efficiency of individual protein fluorescence labeling (calculated according to labeling protocol – see method above).

### Quantification of GST uptake

To compare the rate of uptake of GSTM2, GST-C and various GST-C fragments, the Oregon Green-labeled proteins were added to cells at 200 nM concentration and cells incubated at 37°C/5% CO_2_ for up to 3 hrs. Untreated cells were used for the initial zero time point. Samples were harvested and prepared for flow cytometry at different time points as described above. Mean fluorescence for each GST time point was standardised to the fluorophore to protein ratio for that particular GST, after subtraction of the untreated cell control fluorescence.

### Confocal Laser Scanning Microscopy

Cells were seeded onto no. 1 glass coverslips (Lomb Scientific) in 12-well plates in 10% FBS/RPMI 1640 medium the day before experimentation. Live L-929 cells were used in microscopy experiments to avoid the possibility of artificial localisation. All incubations were performed in serum-free RPMI 1640 medium. Coverslips were rinsed in PBS and viewed in PBS in a heated chamber. Confocal images were obtained with 60× 1.4 N.A. or 100× 1.4 N.A. oil immersion lenses of a Nikon Eclipse TE300 microscope equipped with Biorad Radiance 2000 Laser Scanning system. Excitation was with an argon laser using 515/30 bp emission filters. Data was recorded and analysed using LaserSharp2000 software.

### Circular Dichroism (CD) equilibrium unfolding experiments

GST-C protein fragments were diluted to 4–5 µM (0.1 mg/ml) for CD measurements, and the pH values and solution conditions adjusted to pH 7.2 and 10 mM PO_4_^2−^, respectively. Spectra were recorded on an Applied Photophysics Chirascan spectrometer at 20°C. A cell with a 0.10 cm path length was used for spectra recorded between 190 to 250 nm. The following parameters were employed: spectral bandwidth 1 nm, step size 0.5 nm and time-per-point 0.5 s. Each spectrum was obtained by averaging several scans and the protein CD spectra were corrected for buffer contributions. The temperature was controlled by a Melcor peltier temperature controller. For GST-C denaturing experiments, the molecular ellipticity at 222 nm was recorded at a guanidine HCl concentration range of 0 to 5 M.

Guanidine unfolding curves were fitted to a two-state (N_2_↔2 U) unfolding model (1) [16] using Graphpad Prism and the quality of the fits were assessed by considering the R^2^ value which typically were >0.99.
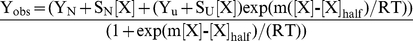
(1)where Y_obs_ is given by the observed ratios mentioned above, and Y_N_, S_N_, Y_U_, S_U_ are intercepts and slopes of the pre-transition and post-transition baselines, [X] is the guanidine concentration, [X]_half_ is the guanidine concentration at the midpoint of transition state, m is the free energy dependence on guanidine concentration and R is the gas constant (1.987×10^−3^). The free energy change from a folded to an unfolded state can be expressed as
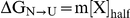



### Differential Scanning Fluorimetry

The thermal stability of GST-C wildtype versus the Y160A and F157A mutants was investigated by thermal denaturation in the presence of SYPRO orange (Invitrogen) [31]. Proteins diluted to 1 mg/ml in pH 7.5 Hepes buffer were mixed 9∶1 v/v with freshly diluted (1∶50, v/v) SYPRO orange. Each protein sample was run in triplicate on an Applied Biosystems 7900HT quantitative real-time PCR instrument using SYBR green settings. Fluorescence was monitored over a temperature gradient of 10–90°C with a 1% ramp rate. Hen egg-white lysozyme (Sigma) was used as a positive control and showed thermal denaturation at 63.4°C, which is comparable to literature values [32].
